# Stressful life events and trajectories of depression symptoms in a U.S. military cohort

**DOI:** 10.1038/s41598-022-14496-0

**Published:** 2022-06-30

**Authors:** Laura Sampson, Howard J. Cabral, Anthony J. Rosellini, Jaimie L. Gradus, Gregory H. Cohen, David S. Fink, Anthony P. King, Israel Liberzon, Sandro Galea

**Affiliations:** 1 Department of Epidemiology, Harvard T.H. Chan School of Public Health, 677 Huntington Avenue, Boston, MA 02115 USA; 2 Department of Biostatistics, Boston University School of Public Health, Boston, MA USA; 3 Center for Anxiety and Related Disorders, Department of Psychological and Brain Science, Boston University, Boston, MA USA; 4 Department of Epidemiology, Boston University School of Public Health, Boston, MA USA; 5 New York State Psychiatric Institute, New York, NY USA; 6 Department of Psychiatry, University of Michigan Medical School, Ann Arbor, MI USA; 7 Department of Psychiatry, College of Medicine, Texas A&M University, College Station, TX USA; 8 Boston University School of Public Health, 715 Albany Street, Boston, MA 02118 USA

**Keywords:** Depression, Epidemiology, Risk factors, Human behaviour

## Abstract

Depression is a common mental disorder that may comprise distinct, underlying symptom patterns over time. Associations between stressful life events throughout the civilian lifecourse—including during childhood—and adult depression have been documented in many populations, but are less commonly assessed in military samples. We identified different trajectories of depression symptoms across four years in a military cohort using latent class growth analysis, and investigated the relationship between these trajectories and two domains of civilian life experiences: childhood adversity (e.g., being mistreated during childhood) and more proximal stressful experiences (e.g., divorce). A four-group depression model was identified, including a symptom-free group (62%), an increasing symptom group (13%), a decreasing symptom group (16%), and a “chronic” symptom group (9%). Compared to the symptom-free group, soldiers with childhood adversity were more likely to be in the chronic depression, decreasing, and increasing symptom groups. Time-varying adult stressors had the largest effect on depression symptoms for the increasing symptom group compared to other groups, particularly in the last two years of follow-up. This study indicates the importance of considering events from throughout the lifecourse—not only those from deployment—when studying the mental health of servicemembers.

## Introduction

Depression is a common and debilitating mental disorder^1^. The majority of epidemiologic studies of depression, particularly among military samples, have assessed prevalence or incidence of dichotomously characterized depression^2–6^. While these studies offer important information regarding factors associated with depression diagnosis, it may be reductive to group different sub-types of depression together into one single outcome considering the heterogeneity of the disorder, the potential for unique symptom profiles within this group, and the fact that depression symptom severity occurs along a continuum^7–10^.

Further, it may be important to understand determinants of sub-threshold depression symptoms, which are often not detected when using dichotomous definitions of depression like those described in the Diagnostic and Statistical Manual for mental disorders (DSM). Sub-threshold depression symptoms can cause substantial functional impairment and may lead to more severe depression symptoms that meet diagnostic criteria, particularly if a stressful life event occurs or worsens^11–13^. Longitudinal studies are useful in helping us to understand why some individuals go on to develop major depression after having sub-threshold symptoms, whereas symptoms subside over time for others, because they allow us to follow the course of symptoms of depression over time, highlighting the natural history of the disorder^14^.

Given the complexities of studying depressive disorders and the advantage of multiple data points over time, group-based latent trajectory methods are useful tools for studying depression longitudinally. These methods have primarily been applied in psychology and other social sciences^15,16^, but are rarely employed in epidemiology. In particular, latent class growth analysis (LCGA) has potential as an approach to examine the growth or shape of trajectories of symptoms over time in a population. LCGA also assesses how members of the population group together in relation to their symptom patterns, and which characteristics predict membership into such groups^17–19^.

There are the three defining characteristics of the current literature on latent trajectories of psychopathology in military and civilian populations. First, most studies among military personnel have modeled posttraumatic stress disorder (PTSD) symptoms, particularly PTSD treatment response^20–22^. Second, most studies of trajectories of psychopathology in general—even those modeling depression symptoms—have anchored symptoms as responses to specific shared events such as psychiatric treatment, pregnancy, natural disasters, or military deployment^23–30^. Third, a large proportion of trajectory studies have focused exclusively on children or adolescents. One review paper describing 25 studies on trajectories of depression symptoms found only four studies of adults (not including elderly adults or other demographically specific populations)^23^, one of which followed symptoms among adults seeking treatment for depression, thus not following the course of any symptom-free respondents at baseline^31^. Similarly, a recent study by Armenta and colleagues followed a military sample over time for PTSD and depression trajectories, among those who screened positive at baseline for both PTSD and major depression, thus not including individuals with sub-threshold or no depression symptoms at the start of follow-up^32^. Taken altogether, there is a lack of studies on trajectories of depression symptoms among adults—and in particular servicemembers—who do not necessarily have a depression history.

It has been long demonstrated that stressful life events are associated with depression^33–39^. Events that occur during childhood may be particularly salient contributors to depression^40,41^; a recent Centers for Disease Control and Prevention report estimated that up to 44% of adult depression in the U.S. may be attributed to adverse childhood experiences^42^. Although we know that traumatic childhood events and more recent adult stressors are associated with depressive disorders^13,34–36,38^, understanding how these events influence specific trajectory group membership and changes in symptom patterns over time can help advance our knowledge. This may be particularly relevant for military populations, as most studies have focused on deployment-related events, neglecting common life stressors and traumas outside of military engagement. No studies to our knowledge have a) examined trajectory classes in a military cohort to describe depression symptoms over time that are not anchored to a specific event (e.g., deployment), and b) assessed the potential impact of traumatic childhood events and time-varying life stressors outside of deployment on such trajectories.

Our aims in this study were to determine (1) how respondents grouped into latent trajectories of depression symptoms across four years of follow-up; (2) the association between traumatic childhood events and membership into these trajectory groups; and (3) the association between ongoing civilian stressors and the course of these trajectories over time, using LCGA in a military cohort.

## Methods

### Data source

We used data from the Ohio Army National Guard Mental Health Initiative (OHARNG-MHI), a cohort study that began in 2008–2009. Data are available upon request at https://www.militarybehavioralhealth.org/contact. All procedures were performed in accordance with the relevant guidelines and regulations.

The details for this study have been described previously^43^. In brief, a study alert letter was mailed to all 12,225 serving National Guard members in Ohio who had current addresses listed with the Guard in 2008. Eight percent of these soldiers (n = 1013) opted out of the study by sending back an opt-out card from the mailing. After allowing time for opt-out responses and removing duplicate entries and soldiers with no working telephone number listed with the Guard (n = 4698), 3980 individuals were contacted before the close of the baseline recruitment year. One-hundred and eighty-seven of these contacted individuals consented to the interview but were then deemed ineligible for the study (e.g., because they were under age 18 or had left the Guard); 31 individuals were disqualified because they did not speak English or had hearing problems; and 1364 declined participation when called. The baseline interview closed at 2616 completed interviews, with an overall response rate of 43% (taking into account all potentially eligible soldiers with working phone numbers), or cooperation rate of 68% (taking into account only those who were successfully contacted before the baseline study’s close). This baseline sample, and the Ohio Army National Guard in general, is representative of the U.S. Army National Guard population as a whole in terms of demographic and social factors such as military rank, gender, and age^43,44^.

This primary cohort was subsequently followed via telephone interviews approximately once per year for the next several years. The interviews were carried out by trained, lay interviewers, and respondents were assured that their responses would be confidential, de-identified, and would have no bearing on the status of their employment with the Guard. Participants gave verbal, informed consent and were compensated for all interviews they completed. The Ohio National Guard and the Boston University Medical Campus institutional review board approved the study protocol.

In order to mitigate loss of sample size over time and related changes in demographics due to attrition and retirement, smaller random samples from newer recruits to the Guard replenished the original group of respondents each year, beginning in the third year of the study, creating a dynamic cohort study design. These participants were enrolled using the same procedures as the primary cohort described above. The second group of participants (following the original cohort) consisted of 578 new respondents whose baseline interviews were conducted during the original group’s third year of the study; the third group of participants included 263 additional respondents whose baseline interviews were carried out during the original group’s fourth year of the study; and the fourth group of participants included 121 respondents whose baseline interviews were conducted during the original group’s fifth year of the study. After the year of their initial baseline interviews, these additional groups’ follow-up interviews were conducted at the same time as and using the same follow-up survey as the other study members from previous groups.

In order to estimate longitudinal depression trajectories, we included participants of the OHARNG-MHI who were present in at least two follow-up waves (among study years 3–6, since the outcome of depression started being counted at wave 3, as described below). Out of 3578 total OHARNG-MHI participants across all cohorts, we excluded 1529 individuals for not having met this criterion. An additional inclusion criterion for the primary cohort in particular was that those individuals were present for the study year 2 interview (wave 2), so that they had data on one of our main exposures of interest, traumatic childhood events (which were only assessed in that year of the study for that cohort). We excluded 205 additional respondents for not having met this additional criterion, arriving at the final analytic sample of 1844.

In all, this analytic sample included participants from the primary cohort (those who enrolled at the beginning of the study in 2008–2009; n = 1207 included in this analytic sample) as well as the first three additional cohorts, who enrolled in 2011–2012 or study year 3 (n = 389 included in this analytic sample), in 2012–2013 or study year 4 (n = 172 included in this analytic sample), and in 2014–2015 or study year 5 (n = 76 included in this analytic sample). Calendar time was used for the time scale in this study, in order to measure trajectories over time. Thus, the earlier years of data for the additional cohorts (e.g., data from study year 2 or 2010–2011 for the first additional cohort) were imputed, as described in the Appendix and depicted in Appendix Fig. 1.

### Exposures

There were two main exposures of interest, both within the domain of civilian life events. The first was exposure to traumatic childhood events, which was assessed once, retrospectively. For the primary cohort, these questions were asked in the second year of the study (during the first follow-up interview). For the remaining groups of participants who entered the study at later years, these questions were included in their baseline surveys. The traumatic childhood event exposure was ascertained using four out of seven questions from the Adverse Childhood Experiences study^45^, including physical, sexual, or emotional abuse by a parent or other adult in the household during childhood, and mental illness, depression, or suicide attempt by a parent or other adult in the household during childhood. Appendix Table 1 lists the four questions included in the surveys. These events were considered time-stable and were used to model trajectory group membership as a binary variable in the primary analysis (trauma compared to no trauma). As a supplementary analysis to assess intensity or severity of childhood trauma, we additionally modeled the childhood trauma variable as a categorical count variable, with the following categories (due to small cell size of having three or four trauma types): no trauma (76.3%), one trauma type (13.6%), and two or more trauma types (10.1%).

The second main exposure of interest included stressful events that occurred more recently in adulthood, such as divorce or serious financial problems (which typically occur in a context outside of military deployment, in civilian life). These events were ascertained at each follow-up interview, asked with reference to events that occurred during the past year (since the last interview). Appendix Table 2 lists the specific stressors that were ascertained. Past-year stressors were used to model the trajectory shape of depression symptoms at each wave. This variable was dichotomized (i.e., presence of one or more stressors each year), given the small cell size that resulted from assessing specific individual events.

In order to preserve temporality and reduce the potential for reverse causation in between waves (e.g., incident depression may cause absenteeism and indirectly lead to job loss which is considered a stressor), past-year stressors were lagged as follows: stressors that occurred between year 1 and year 2 (assessed at year 2) were used to model depression symptoms between years 2 and 3 (assessed at year 3), and so on through wave 6. Consequently, the outcome was measured beginning in study year 3, describing symptoms that occurred between years 2 and 3.

### Confounders

Potential confounders were chosen based on previous literature and hypothesized associations with depression symptom trajectories. For the traumatic childhood events exposure, we included the following potential confounders: biological sex (female and male); age at baseline, categorized into 18–24 years, 25–34 years, and 35+ years based on survey response options; and self-reported race and ethnicity, dichotomized into two categories for analyses due to small cell size of most racial and ethnic groups. The categories were White compared to: Asian, Black or African American, Native Hawaiian or Other Pacific Islander, Hispanic, or “other” race or ethnicity as listed on the survey instrument. These variables were all time-stable and assessed at baseline.

Potential confounders considered for the time-varying adult stressor exposure (assessed in a separate model from the one described above) included the same time-stable baseline confounders described above, although age was further collapsed to be 25+ years compared to 18–24 years, given small cells with more variables added. Additionally, we controlled for the following variables that might further confound the relationship between past-year stressors and depression symptoms: marital status at baseline (married vs. not); past-year total household income at baseline (categorized into $40,000 or less, $40,000-$80,000, and more than $80,000, based on survey response options); educational attainment at baseline (categorized into high school degree or less, some college or technical training, and college degree or more); military rank at baseline (Enlisted, Officer, Warrant Officer, and other); and the following lagged time-varying confounders: past-year deployment (deployed, not deployed) and past-year PTSD, defined by meeting DSM-IV criteria^46^ using the PTSD CheckList—Civilian version^47^.

### Outcome

Depression was operationalized as a count of the number of endorsed symptoms in the past month at each follow-up wave, if the symptom occurred for at least a two-week period and at least “more than half the days” within that period, from a modified version of the Patient Health Questionnaire–9 (PHQ-9)^48^. This modified questionnaire first asked participants whether and how often they experienced each PHQ-9 symptom with reference to their entire lifetime (at the baseline interviews) or “since the last interview” (at the follow-up interviews, with the date of the last survey provided). Respondents who endorsed ever experiencing one or more symptoms (either since the last interview or at some point in their life, depending on the study year and cohort) were then asked whether or not each symptom was present in the past 30 days (yes/no questions). Thus, in order to a) assess current symptoms, given the data structure of our dynamic cohort which results in different time scales being used for different cohorts at the same time, and b) ensure that the reported individual symptoms occurred temporally close together to each other, we used the binary past-month symptom questions rather than the full scale. Accordingly, each symptom was coded as a dichotomous 0/1 variable before being summed (range: 0–9 symptoms).

### Statistical analysis

First, after the exclusions described in the data source section above, we performed single imputation on missing data within the analytic sample using multivariable regressions with fully conditional specification, otherwise known as imputation by chained equations^49,50^. See the Supplementary Methods and Appendix Fig. 1 for more information on the imputation. One variable was not able to be imputed in this way, as there were no non-missing values: past-year stressors were not assessed during the 2014–2015 interview year due to a shorter survey that year. The variable for presence of past-year stressors at wave 5 was thus handled using the maximum likelihood method within the PROC TRAJ procedure in SAS described below, as is the default within this procedure^51^.

After imputation, we ran descriptive analyses to check the data and assess the distribution of variables (Table 1). For the outcome of number of depression symptoms at each time point, we determined that the zero-inflated Poisson distribution—a generalization of the standard Poisson distribution—was appropriate for the semi-parametric LCGA, as our outcome was a count of symptoms and the majority of individuals had 0 symptoms at each time point^51,52^.

**Table 1:** Sample characteristics: frequencies of confounders and exposures of interest (n = 1844).

	n	%
**Time-stable variables**		
Biological sex		
Male	1573	85.3
Female	271	14.7
Age group		
Age 18–24	797	43.2
Age 25–34	532	28.9
Age 35 +	515	27.9
Self-reported race and ethnicity		
White race	1618	87.7
Asian, Black or African American, Native Hawaiian or Other Pacific Islander, Hispanic, or “other” race or ethnicity	226	12.3
Marital status at baseline		
Currently married	750	40.7
Not currently married	1094	59.3
Total household income at baseline		
Less than or equal to $40,000	728	39.5
More than $40,000 to $80,000	674	36.5
More than $80,000	442	24.0
Education at baseline		
High school degree or less	451	24.5
Some college or technical training	900	48.8
College degree or more	493	26.7
Rank at baseline		
Enlisted	1537	83.4
Officer	252	13.7
Warrant Officer	25	1.4
Other	29	1.6
Traumatic childhood event experience		
One or more traumatic childhood events	437	23.7
No traumatic childhood events	1407	76.3
**Time-varying variables**		
Past-year deployment (average across follow-up waves)		
Deployed in the past year	243	13.2
Not deployed in the past year	1601	86.8
Past-year PTSD (average across follow-up waves)		
Past-year PTSD	85	4.6
No past-year PTSD	1759	95.4
Past-year stressor experience (average across follow-up waves)		
One or more stressors per year	911	49.4
No stressors per year	933	50.6

PTSD = posttraumatic stress disorder. One participant was missing on rank and was not included in the percentages for that variable.

**Figure 1:**
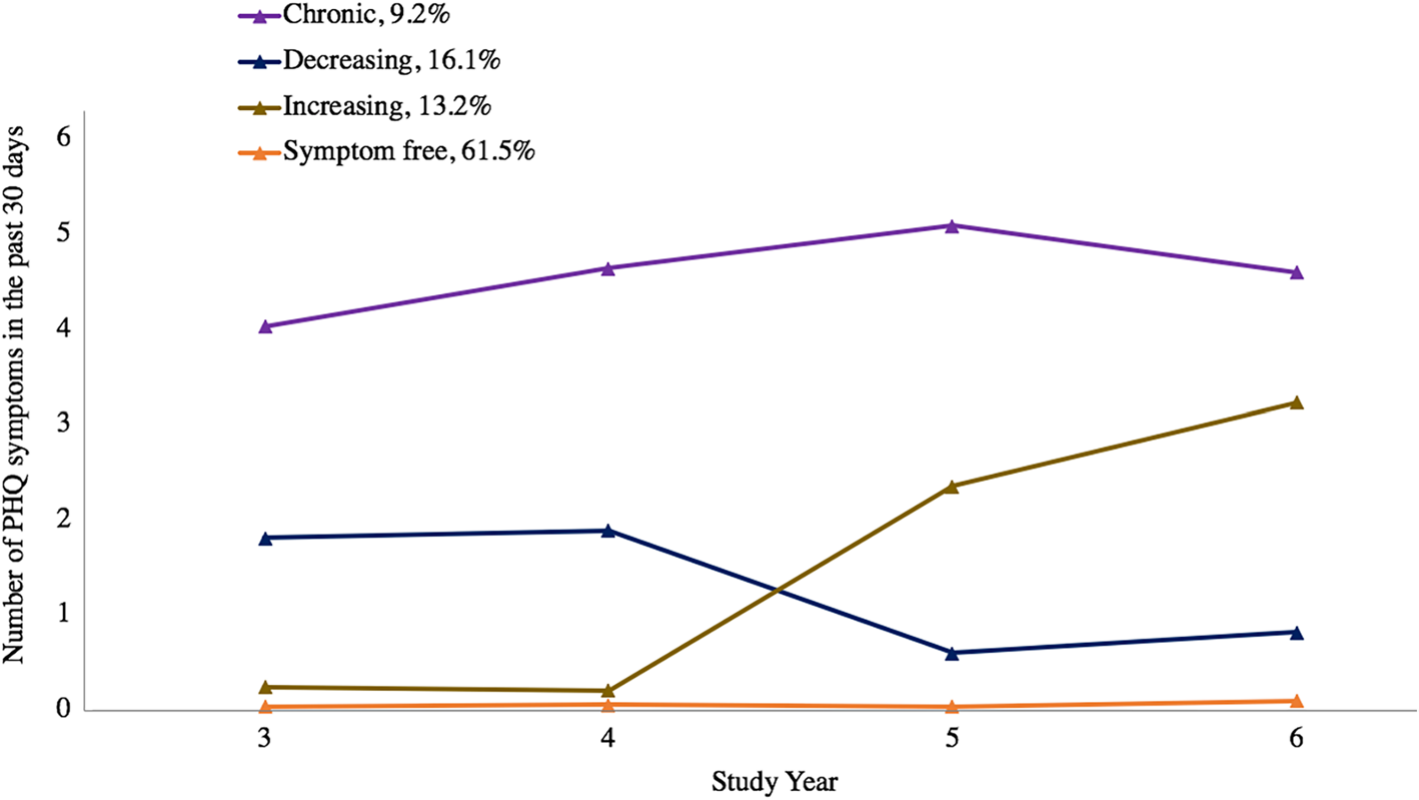
Latent trajectories for number of PHQ-9 depression symptoms in the past 30 days across four follow-up years, modeled using a zero-inflated Poisson distribution (n = 1844). PHQ = Patient Health Questionnaire (nine total symptoms).

We then applied LCGA, which identifies latent homogeneous groups within a larger population using the distribution of the outcome data over time (based on slopes and intercepts), with the PROC TRAJ procedure in SAS^51,53^. We fit different numbers of trajectory groups and different functional forms for each group—making only one change at a time—in a stepwise fashion, until an optimal model was chosen. The Bayesian Information Criteria (BIC), Akaike Information Criterion, average posterior probabilities for membership into each group, model convergence, and visual differentiation between the different groups were all considered together to estimate the ideal number of trajectory groups and best-fitting model^51,54^. Table 2 shows fit statistics for all of the trajectory models tested; the model with two asterisks represents the chosen model. We then plotted the final model’s trajectories using the average predicted symptoms of each group (Fig. 1).

**Table 2:** Fit statistics for latent class growth analysis of number of depression symptoms in the past 30 days, modeled using a zero-inflated Poisson distribution with different numbers of groups and functional forms (n = 1844).

	Functional form (0 = intercept only, 1 = linear, 2 = quadratic, 3 = cubic)											
Number of groups	Group 1	Group 2	Group 3	Group 4	Group 5	BIC	AIC	Average predicted probability of group 1	Average predicted probability of group 2	Average predicted probability of group 3	Average predicted probability of group 4	Average predicted probability of group 5
2	3	3				− 6849.51	− 6824.67					
2	3	2				− 6847.52	− 6825.44					
2	2	2				− 6844.02	− 6824.70					
2	2	1				− 6841.42	− 6824.86					
2*	1	1				− 6838.60	− 6824.80	0.98	0.97			
3	3	3	3			− 6524.44	− 6485.80					
3	3	3	2			− 6520.77	− 6484.89					
3	2	3	2			− 6517.17	− 6484.05					
3	1	3	2			− 6513.42	− 6483.06					
3	1	2	2			− 6511.29	− 6483.69					
3*	0	2	2			− 6509.02	− 6484.18	0.95	0.87	0.92		
4	3	3	3	3		− 6304.33	− 6251.90					
4**	3	3	3	2		− 6300.57	− 6250.90	0.93	0.84	0.82	0.93	
5	3	3	3	3	3	− 6264.70	− 6198.46					
5	3	3	3	3	2	− 6261.02	− 6197.55					
5	2	3	3	3	2	− 6259.63	− 6198.91					
5	2	2	3	3	2	− 6255.94	− 6197.98					
5	2	1	3	3	2	− 6252.49	− 6197.29					
5*^	2	0	3	3	2	− 6249.12	− 6196.68	0.74	0.91	0.81	0.82	0.92

BIC = Bayesian Information Criterion.

AIC = Akaike Information Criterion.

* = best model for the number of groups, for which latent probabilities (average predicted probabilities of group membership) were assessed for each group.

^ = increasing group split into two non-distinct groups for this model.

** = best overall model/final chosen number of groups.

Once the optimal model was chosen, we used the two traumatic childhood event exposures (binary and count variable) in crude and adjusted multinomial logistic regression models, where a categorical variable representing the four trajectory groups was modeled as the outcome, with the symptom-free group as the reference category (Table 3 and Appendix Table 3).

**Table 3:** Crude and fully adjusted multinomial models for the associations between reporting one or more traumatic childhood events and membership into each depression symptom trajectory group (n = 1844).

	Crude		Adjusted	
	OR	95% CI	OR	95% CI
Symptom-free group (referenc﻿e)				
Decreasing group	2.41	(1.81, 3.21)	2.33	(1.75, 3.11)
Increasing group	1.81	(1.32, 2.50)	1.78	(1.29, 2.45)
Chronic group	3.82	(2.72, 5.37)	3.57	(2.53, 5.05)

OR = odds ratio.

CI = confidence interval.

Controlling for biological sex, age group, and self-reported race and ethnicity.

The second exposure of interest (one or more stressful events in the year prior to each depression assessment) was time-varying and thus added to the LCGA models directly, in order to model changes in the symptom patterns themselves, instead of predicting trajectory group membership which is standard for time-stable risk factors^51^. We first ran a crude stressor model, followed by an adjusted model.

**Figure 2:**
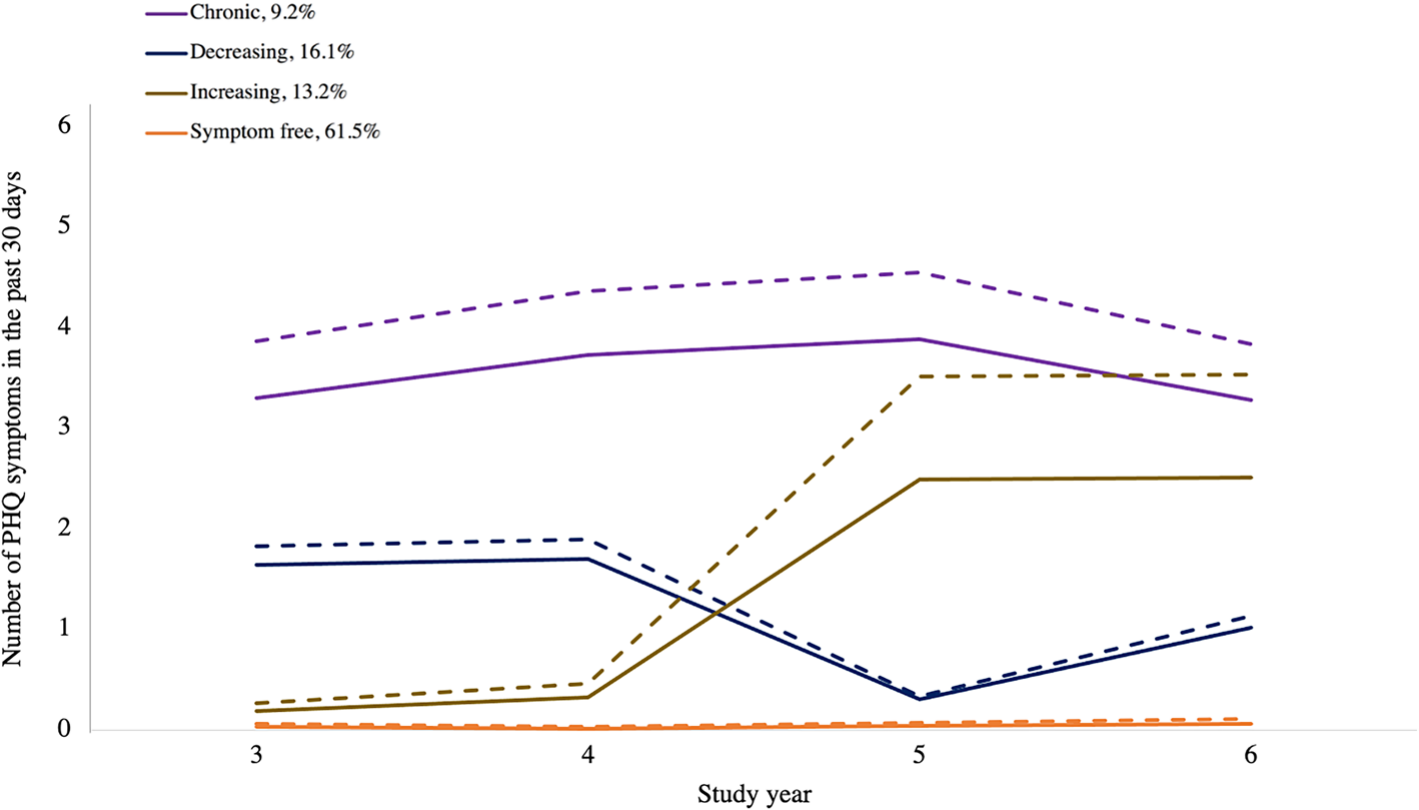
Latent trajectories for number of PHQ-9 depression symptoms in the past 30 days across four follow-up years, modeled using a zero-inflated Poisson distribution, with and without past-year stressors at each follow-up year while holding PTSD constant (n = 1844). Dotted line = one or more past-year stressors at every time point, but no past-year PTSD or deployment at any time point, to isolate the potential effect of stressors. Solid line = no stressors, PTSD, or deployment in the past year at any time point. PHQ = Patient Health Questionnaire (nine total symptoms). PTSD = posttraumatic stress disorder. Controlling for biological sex, age group, self-reported race and ethnicity, marital status, income, education, rank, time-varying PTSD, and time-varying deployment experience. One participant was excluded from this model due to missing rank.

Finally, we used results from the plottcov function in PROC TRAJ to plot the average predicted symptoms of each trajectory when setting the time-varying covariates to fixed values^52^. In order to isolate the potential effect of stressors on depression symptoms at each time point, we fixed past-year PTSD and past-year deployment (the two time-varying confounders) as being equal to 0 at all time points (the most common values), and compared the number of depression symptoms at each time point and overall shape of the trajectories when past-year stressors were set to be equal to 1 compared to 0 at each year (Fig. 2).

### Sensitivity analyses

As a sensitivity analysis, the steps described above were repeated among individuals from the primary cohort who had no lifetime history of depression at wave 2 or earlier (prior to the start of follow-up time at wave 3), in order to assess whether the relationships we found in our main analyses held when reducing the likelihood of reverse causation between our exposures and depression symptoms (e.g., prior depression making subsequent stressors more likely to occur, through stress generation^55,56^). (See Appendix for results).

#### Quantitative bias analysis

As an additional sensitivity analysis, we ran a simple quantitative bias analysis to estimate the potential impact of differential recall of childhood events by depression status in the main analysis (Appendix Table 6). To do this, we used the exposure misclassification spreadsheet within the “Simple Sensitivity Analyses for Misclassification” workbook, based on the methods described in the quantitative bias analysis textbook by Lash and colleagues^57^. We used the crude pairwise comparisons of both the chronic symptom group and the decreasing symptom group—the two groups that had depressive symptoms at the beginning of follow-up and thus most likely to have had depression symptoms at the time of the childhood event ascertainment—each compared with the symptom-free group. We then varied the sensitivity and specificity of correct ascertainment of having the childhood event exposure, ranging from 70 to 90% sensitivity and 90% to 99% specificity as plausible values, where the sensitivity of exposure ascertainment among those in the higher depression symptom trajectory groups was always higher than those in the symptom-free group, and the specificity of exposure ascertainment among those in the higher depression symptom trajectory groups was either equal to or lower than those in the symptom-free group (which we expected to be the most common patterns given no gold standard assessment comparison).

## Results

### Descriptive results

Table 1 shows sample characteristics and prevalence of all variables used. The majority were male (85.3%) and White (87.7%). Forty-three percent were between the ages of 18–24; about 29% were between the ages of 25 and 34, and 28% were 35 or older. Almost 24% reported one or more traumatic childhood events. On average—per year during follow-up—just under half of the sample reported one or more stressors.

### Trajectories

Table 2 shows the fit statistics for different potential trajectory models. A four-group model (with three trajectory groups fit with cubic terms and one with a quadratic term) was chosen as the overall best fitting model, with a BIC value of -6300.57 and average predicted probabilities of group membership ranging from 82 to 93% per group.

As can be seen in Fig. 1, the groups from this model included a stable, symptom-free group (showing essentially no symptoms at any point during follow-up, 61.5% of the sample); an increasing depression symptom group (13.2%, going from on average no depression symptoms at the first follow-up year to about 3 depression symptoms in the last follow-up year); a decreasing depression symptom group (16.1%, going from about 2 depression symptoms at the first year of follow-up to 1 or no depression symptoms at the last year of follow-up); and a “chronic” depression symptom group (9.2%, fit with the quadratic slope, staying essentially steady around 4–5 symptoms throughout follow-up).

### Childhood events

Table 3 shows adjusted odds ratios (aOR) for the associations between reporting one or more traumatic childhood events and membership into each trajectory group, with the symptom-free group as the reference category, from crude and adjusted multinomial models. After controlling for biological sex, age, and self-reported race and ethnicity, those who reported childhood events had 3.57 times the odds (95% confidence interval (CI) 2.53, 5.05) of belonging to the chronic depression symptom group compared to the symptom-free group. Reporting childhood events was also associated with being in the decreasing and increasing depression symptom trajectory groups compared to the symptom-free group (aOR 2.33, 95% CI 1.75, 3.11 for the decreasing group and aOR 1.78, 95% CI 1.29, 2.45 for the increasing group).

Appendix Table 3 shows the same model but using a three-level count variable of number of childhood trauma types instead of a binary exposure variable. The same pattern was observed, with the highest odds ratios seen for the chronic and decreasing symptom groups. In particular, the highest odds ratios were observed for respondents with two or more trauma types, suggesting that a higher severity or occurrence of childhood trauma is associated with higher-symptom trajectory groups. In particular, those with two or more trauma types were over five times more likely to belong to the chronic depression symptom group, compared to those with no trauma (aOR: 5.32, 95% CI: 3.43, 8.27 for the chronic group compared to the symptom-free group).

### Adult stressors

Figure 2 shows the plotted latent trajectories, like Fig. 1, but comparing two versions for each group: one showing the trajectories when modelling 0 past-year stressors reported at all time points (solid lines) and one with 1+ past-year stressors reported at every time point (dotted line), while adjusting for confounders. In both versions, lagged, past-year PTSD and deployment were held constant at 0 for each time point (the most common values), in order to isolate the potential effect of time-varying stressors. Stressors had the largest effect on depression symptoms for the increasing depression symptom group, particularly in years 5 and 6 (where there was a difference of 1.02 symptoms at each year, for stressors compared to no stressors), suggesting that the potential influence of stressors on these symptoms might depend on the underlying pattern or number of symptoms. The decreasing and symptom-free groups saw a very small—potentially negligible—change in symptoms (with an average difference over time of about 0.13 depression symptoms and 0.03 depression symptoms, respectively), while an average increase of 0.61 symptoms was seen for the chronic depression symptom group, mostly unchanging across the follow-up time.

### Sensitivity analysis

See Appendix for sensitivity analysis results, including quantitative bias analysis results.

## Discussion

We identified four distinct latent trajectory groups of depression symptoms across four years of follow-up in a cohort of Army National Guard servicemembers. Reporting traumatic childhood events—especially two or more different types of childhood trauma—was strongly associated with having chronic, decreasing, or increasing numbers of depression symptoms over time, compared to having consistently no symptoms. Additionally, reporting one or more stressors during follow-up was associated with an increase in depression symptoms among all trajectory groups.

The majority of persons in this study fell into the symptom-free group, suggesting that they are likely psychologically resilient^58^ despite high exposure to stressful and traumatic events, in and outside of military engagement. This finding is consistent with many other studies of trajectories of psychopathology over time, either after specific events^21,25,29^ or in general over time^23,59,60^, supporting the overall idea that individuals tend to be modally resilient in the face of stress^19^.

The four trajectory groups detected in the present study are consistent with the four groups found in a prior study done with the same underlying cohort but only using data from study years 1–4 and a smaller subsample of soldiers who deployed within the two years before baseline, with a timescale of time since deployment^29^. Further, although the review paper by Musliner and colleagues on studies of trajectories of depression included primarily studies of children, adolescents, and older adults, the overall summary of trajectory group results they described were similar to ours; most studies reviewed found only a minority proportion of respondents who fell into trajectories with persistent depression symptoms over time, and the studies that specifically modeled trajectories among adults each detected 3–4 trajectory groups, including a high depression symptom group, a low depression symptom group, and then either a moderate depression symptom group or some combination of intermittent, increasing, and/or decreasing depression symptom groups^23^. A study by Armenta and colleagues—although conducted only among servicemembers and veterans who had comorbid PTSD and major depression at baseline—also identified four distinct trajectory groups, including a chronic symptom group similar to ours^32^.

Respondents in our study who experienced one or more traumatic childhood events were more likely to have a higher number of symptoms over time compared to those with no childhood trauma. This finding is consistent with a recent study on trajectories of depression symptoms among Danish soldiers after a deployment^30^, and with a study among post-partum women in France^61^, despite the differences in population types. Considering our knowledge of the relationship between trauma and depression outcomes in general^5,34–38^, it is not surprising that these events would be associated with chronic or increasing symptoms over time. However, it is potentially surprising that adverse childhood events were also associated with membership in the decreasing depression symptom groups in this study. We observed higher magnitudes of effect for the decreasing groups in comparison to the increasing depression symptom groups (albeit with overlapping confidence intervals), but both were modeled with reference to the symptom-free group. It is likely that there are additional factors contributing to the decreasing depression symptom groups’ trajectories that we were unable to measure, including modification or mediation by factors such as treatment for depression. Further, several years have elapsed between the presumed time of the childhood events and the time of the first follow-up of depression symptoms, during which many other unobserved events and characteristics may have affected these patterns. Future research should further investigate this decreasing depression symptom group.

Our findings were also consistent with other trajectory studies that incorporated life stressors into their analyses, in that others have found stressors to be associated with higher-symptom trajectory groups^29,31,60^. However, to our knowledge all of these studies used presence of past stressors as a time-stable risk factor, thus only predicting trajectory group membership, not modelling change in depression symptoms over time, as we did with time-varying stressors. Consequently, it is difficult to compare our results for past-year stressors with other similar studies.

When we modelled past-year stressors at each time point, the increasing depression symptom groups showed the largest increase in symptoms compared to the other groups, suggesting that the potential influence of stressors on these symptoms might depend on the underlying pattern or number of symptoms. This finding may also be at least partially explained by reverse causation, with increasing depression symptoms leading to more stressful events, as explained by the stress generation phenomenon^55,56^. This potential mechanism is supported by our sensitivity analysis among individuals with no history of depression at baseline, where the change in depression symptoms was smaller among the increasing depression symptom group, as compared to the main analyses. By contrast, in this sensitivity analysis, the chronic depression symptom group saw the largest increase in symptoms overall. However, it is difficult to compare the two analyses directly, given the inherent differences in individuals with no history depression, including the overall shape of their trajectories.

One limitation of this study is the influence of potential reciprocity among some of the relations studied, as described above. For example, despite the fact that past-year stressors were lagged to describe the year before the current depression outcome, initial depression symptoms which were present at baseline may indirectly cause a stressor such as job loss to occur in the future, instead of the stressor causing future or persistent depression. The idea that depression can lead to stressful events in a person’s life has been previously described by the stress generation phenomenon^55,56^, which suggests that a depressed individual’s characteristics and behaviors can strain interpersonal relationships, in turn causing stressful events such as divorce, break-ups, or job loss. However, this limitation of our study is offset by our ability to follow not only the symptom path of respondents who develop depression over time but also the symptom path of those who have chronic, persistent, or relapsing/remitting depression. Further, our sensitivity analyses, which subset all analyses to respondents who had no prior history of DSM-IV depression at the start of follow-up, produced similar results for all aspects of the analyses, suggesting that our results are unlikely to be fully explained by reverse causation. It remains possible, however, that sub-threshold symptoms prior to the start of follow-up may have increased the likelihood of stressful events occurring.

A second limitation is the potential for misclassification of variables. One potential pattern of misclassification is differential recall of traumatic childhood events by current depression status at the time of event ascertainment. For example, respondents who were depressed at the beginning of the study may have been more likely to remember or report mistreatment during childhood. Thus, depressed individuals may be more likely to have higher sensitivity and lower specificity of exposure classification. We attempted to correct for these potential patterns of differential misclassification using a simple bias analysis, and found that although this did attenuate the associations, the misclassification is unlikely to account for the entirety of the relationships, given the parameters we assumed. Further, prior literature has suggested that mental health status at the time of reporting childhood abuse may not actually affect ascertainment or recall of events^62^. However, our calculations do not account for dependent error (or “same source” bias), which is likely to further bias our results away from the null, because reporting depression symptoms may be related to reporting childhood events or stressors^63^. Nonetheless, even if events and symptoms were misclassified due to poor recall or incorrect reporting, perceived mental health symptoms and perceived life events are of clinical interest both in the literature and to the military, as they affect functional health and predict retention and performance in the military^64^.

Third, dichotomizing the ongoing adult stressors may have resulted in arbitrary grouping of experiences. Future studies, if sample size allows, should aim to compare the effects of different cut-offs for number of stressful events, or test specific types of events individually.

Despite inherent limitations, this study capitalized on the full range of depression symptoms over time, elucidating different subgroups and patterns of depression symptoms in a U.S. military cohort. Our study was the first, to our knowledge, to show that recent events with which soldiers may struggle in civilian life, such as financial problems or divorce, may increase depression symptoms over time, particularly among individuals who already have a high level of symptoms. These patterns may not be discernable when only using dichotomous diagnoses of depression. Further, our findings indicate that childhood events may have effects on depression symptoms many years after they first transpired, highlighting the importance of considering traumatic and stressful events that occur throughout the entire lifecourse when studying the mental health of military personnel.

The relationships between these types of events and depression in general have been explained in other populations using psychosocial frameworks. For example, the Conservation of Resources theory—first posited by Stevan Hobfoll—explains that the accumulation and retention of resources such as money, housing, and social networks act as buffers for mental health problems, including depression^65^. When those resources are threatened or lost—through events such as divorce and job loss—stress typically occurs, which can then lead to depression through the lack of formerly protective buffers. Further, stressful events rarely occur in isolation; many have theorized that “loss begets loss” and “stress begets stress”^66,67^. Thus, one stressor may cause others to occur, making eventual depression even more likely.

Although our findings should be replicated in additional samples, they may have important policy implications. For example, differences in treatment of depression may be considered for servicemembers who have experienced traumatic childhood events compared to those who have not. Specifically, individuals with complex histories of trauma may respond better to combinations of medication and therapy compared to medication alone. Additionally, National Guard leadership may wish to focus on modifiable factors such as social support within units and psychological resources for coping with stress as potential mediators that could serve as buffers in the relationship between traumatic childhood events and eventual depression^13^. In terms of ongoing stressors such as relationship problems or civilian job loss, the Guard may wish to offer programs such as family support, job training, or housing support, some of which the Ohio Army National Guard has already begun to initiate. Despite cost and logistical barriers, these types of interventions may be important to consider for soldiers’ mental health, particularly given their frequent switch between civilian and military engagement.

## Supplementary Information

Supplementary Information.
