# Supplementary material for: Stressful life events and trajectories of depression symptoms in a U.S. military cohort
Source: Sci Rep. 2022 Jun 30;12:11026. doi: 10.1038/s41598-022-14496-0 (PMC9246834; doi:10.1038/s41598-022-14496-0)
Supplement: Supplementary file 1 — Supplementary Information. [file 41598_2022_14496_MOESM1_ESM.docx]

**SUPPLEMENTARY METHODS**

**Imputation:**

We performed single imputation on missing data within the analytic sample, using multivariable regressions with fully conditional specification, otherwise known as imputation by chained equations within PROC MI in SAS. Imputation by fully conditional specification is flexible because it can be used with arbitrary missing data patterns and is also ideal for imputing categorical variables, as logistic regression can be used for binary and ordinal variables. This method imputes variables in order of missingness, such that the maximum amount of information is used at all times. The variables with the least amount of missingness are imputed first, followed by those with the most amount of missingness. This is done so that the variables with a higher number of missing values are imputed using the recently imputed values of other variables that had fewer missing values and thus relied less on imputation, if those variables are applicably predictive (in addition to using other variables that are predictive but which did not have missing data). The procedure starts with 20 burn-in iterations.

We ran all analyses both with and without the imputation, and the non-imputed results were almost identical and thus are not included here, but are available on request.

As shown below, the amount of missingness in the original dataset ranged from only one respondent missing on some variables assessed at baseline (due to responding “don’t know” or refusal to answer those questions), to 716 respondents (across all cohorts) missing on presence of past-year stressors at study year 3. There were more missing values on the time-varying variables at the beginning of follow-up (e.g., study years 2 and 3) given the fact that the additional cohorts who started in study years 3 and 4 did not yet have past-year ascertainment at the early waves and were thus imputed in order to be included in analyses (in addition to members of the primary cohort who had been lost to follow-up by that time). See Appendix Figure 1 for additional information.

Imputation was used for the following variables, in order of missingness:

- Childhood trauma (n=1 respondent missing, or 0.05% of the sample)
- Marital status at baseline (n=1, 0.05%)
- Rank at baseline (n=1, 0.05%)
- Race at baseline (n=5, 0.27%)
- Income at baseline (n=50, 2.7%)
- Past-year depression at study year 3 (n=415, 22.5%)
- Past-year deployment at study year 3 (n=415, 22.5%)
- Past-year PTSD at study year 3 (n=419, 22.7%)
- Past-year depression at study year 4 (n=447, 24.2%)
- Past-year deployment at study year 4 (n=448, 24.3%)
- Past-year PTSD at study year 4 (n=450, 24.4%)
- Past-year depression at study year 6 (n=464, 25.2%)
- Past-year deployment at study year 5 (n=507, 27.5%)
- Past-year PTSD at study year 5 (n=507, 27.5%)
- Past-year depression at study year 5 (n=507, 27.5%)
- Presence of past-year stressors at study year 4 (n=599, 32.5%)
- Presence of past-year stressors at study year 2 (n=637, 34.5%)
- Past-year PTSD at study year 2 (n=637, 34.5%)
- Past-year deployment at study year 2 (n=638, 34.6%)
- Presence of past-year stressors at study year 3 (n=716, 38.8%)

**SUPPLEMENTARY RESULTS**

**Sensitivity analysis results**

Appendix Table 4 shows the fit statistics for different trajectory models among the subsample of individuals in the primary cohort who had no history of depression prior to the start of follow-up (n=880). The model with three trajectory groups and the model with four trajectory groups both appeared to be good fits.

The three-group model, shown in Appendix Figure 2, was similar to those seen in the main analyses, except the increasing and decreasing groups were essentially combined into one stable, mild group (likely due to the smaller sample size), with about 1 symptom throughout follow-up. As expected, the overall number of symptoms for all groups was lower than seen in the primary analyses, since those with any history of diagnosable depression were excluded.

The four-group model, shown in Appendix Figure 3, was more similar to those seen in the main analyses, and had extremely similar distributions of individuals who fell into each latent group, with the main difference being the smaller number of symptoms at the start of follow-up time, such that the “chronic” depression symptom group was more of an increasing-to-chronic depression symptom group, and the increasing and decreasing depression symptom groups were also at lower levels overall. Again, these differences are consistent with the fact that this sample had no history of DSM-IV depression prior to follow-up time, and thus is much less likely to start with a high number of symptoms. There was also a slightly higher proportion of individuals with no symptoms over the four years (65.5%) compared to the full sample, which is also consistent with the fact that those who previously had any depression were removed.

For the models incorporating traumatic childhood events and time-varying stressors, we used the four-group model, in order to more directly compare results to the main analyses. Appendix Table 5 shows odds ratios for the associations between one or more traumatic childhood events and membership into each trajectory group, from crude and adjusted multinomial models. All odds ratios were elevated and followed the same general pattern as those in the main analyses, but were attenuated: those who reported childhood events had 2.15 times the adjusted odds (95% CI: 1.24, 3.73) of belonging to the chronic depression symptom group compared to the symptom-free group. Adjusted odds ratios were 1.49 (95% CI: 0.91, 2.44) and 1.89 (95% CI: 1.14, 3.16) for the decreasing and increasing trajectory groups, respectively, when both compared to the symptom-free group. Due to the smaller sample size in this sensitivity analysis, confidence intervals were larger and thus the estimates were less precise than the full sample main analyses.

Finally, Appendix Figure 4 shows the plotted latent trajectories with no past-year stressors reported at any time point compared to one or more past-year stressors reported at every time point. The changes in symptoms followed the same pattern as in the main analyses, but the chronic depression symptom group saw a slightly larger increase in symptoms (an average of 1.1 symptoms across the four time points), while the increasing depression symptom group saw a smaller increase in symptoms compared to the main analysis (an average of 0.2 symptoms across the four time points), though still increasing across time, peaking at study year 5 with a difference of 0.4 symptoms. As with the main analyses, the symptom-free group also saw a very small (essentially negligible) but stable difference of 0.04 symptoms, and the decreasing depression symptom group had an average difference of 0.6 symptoms over time. In this model including time-varying covariates, however, the “decreasing” depression symptom group changed shape slightly, increasing in symptoms at the last time point (for both the stressor and non-stressor groups), which may be due to the overall smaller sample size and lower precision in this model, as there appears to be more overlap between groups, with crossing lines toward the end of follow-up.

#### Quantitative bias analysis results

Appendix Table 6 shows results from the simple quantitative bias analysis, varying different potential sensitivity and specificity values for childhood event ascertainment, which differ based on trajectory group membership (in each case, “depression+” refers to being in the higher symptom group in each comparison, whereas “depression-” refers to being in the symptom-free group). For the decreasing group compared to the symptom-free group, the uncorrected, crude OR for one or more childhood events was 2.41, as described in the main results. When varying the sensitivity and specificity of childhood event ascertainment, the crude, corrected OR ranged from 2.21 to 1.63, assuming a valid bias model, where the lowest value was that with 90% sensitivity and 95% specificity of exposure ascertainment among those in the decreasing depression symptom group (the higher symptom group at baseline in this comparison), and 70% sensitivity and 99% specificity among those in the symptom-free group. For the comparison of the chronic depression symptom group with the symptom-free group, the uncorrected OR was 3.82, and the corrected OR’s ranged from 3.67 to 2.80, assuming a valid bias model, where the lowest value had the same combination of sensitivity and specificity values as in the previous comparison.

Appendix Figure 1. Diagram of imputed data by cohort. ^a^


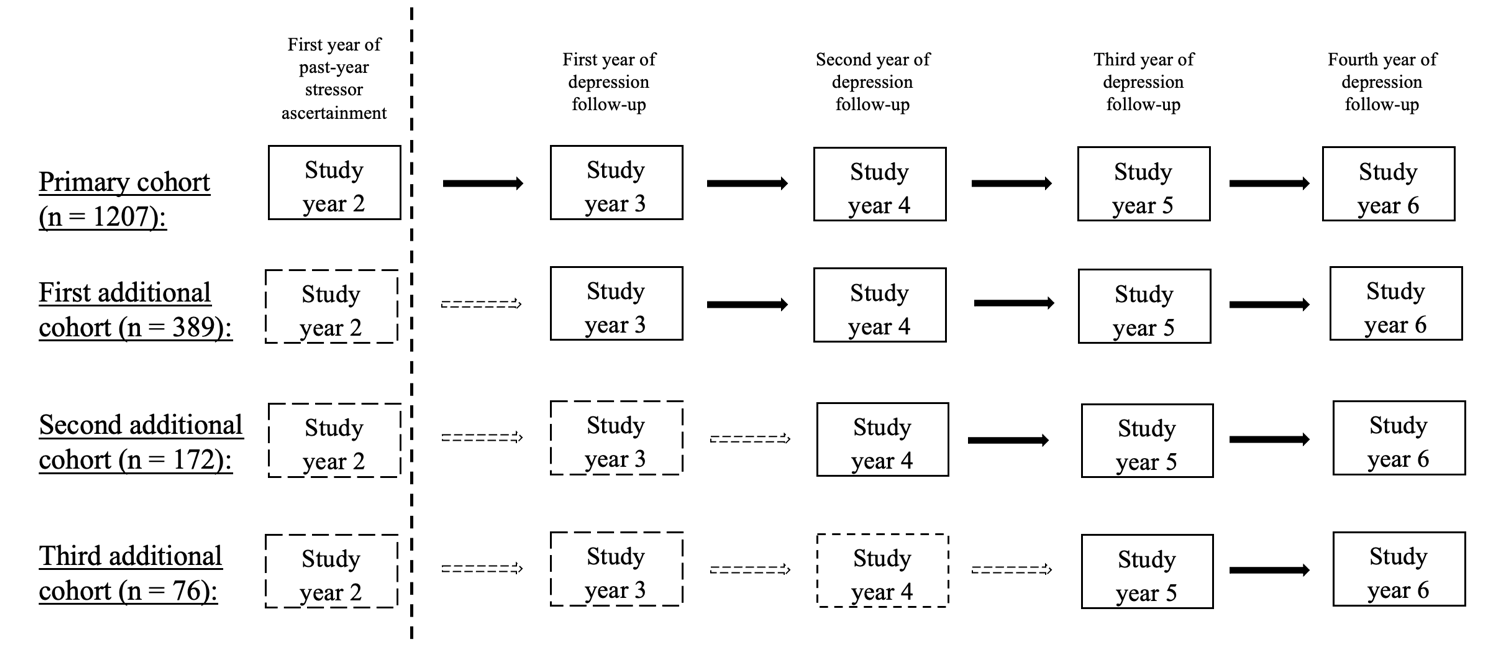


^a^ Dashed boxes = fully imputed data (other than time-stable confounders assessed at each cohort’s baseline interview, e.g., race, biological sex).

Appendix Table 1. Traumatic childhood event questions in the OHARNG-MHI survey.

| *Did a parent or other adult in the household you grew up in* often or very often insult you, put you down, or act in a way that made you afraid you would be physically hurt? |
| --- |
| *Did a parent or other adult in the household you grew up in* often or very often grab, shove, or slap you, or hit you so hard you had marks or were injured? |
| *Did a parent or other adult in the household you grew up in* touch you or have you touch them in a sexual way, or attempt to or actually have oral, anal, or vaginal intercourse with you? |
| *Was a parent or other adult in the household you grew up in* depressed or mentally ill, or ever attempt suicide? |

Appendix Table 2. Stressful event questions asked each year in the OHARNG-MHI follow-up surveys (and at baseline surveys with reference to lifetime experience).

| Since we last spoke in [MONTH] or [YEAR], have you… |
| --- |
| Lost a job, been laid off, or lost a large part of your income? |
| Lost your house that you owned or were homeless? |
| Been robbed or had your home broken into? |
| Been unemployed and seeking employment for at least three months? |
| Had serious financial problems? |
| Experienced stressful legal problems – for example, being sued or suing someone else? |
| Had a family member with a serious drug or alcohol problem? |
| Been through a divorce or “break up” with a partner or significant other? |
| Been emotionally mistreated – for example, shamed, embarrassed, ignored, or repeatedly told you were no good? |

Appendix Table 3. Crude and fully adjusted multinomial models for the associations between number of types of traumatic childhood events compared to no trauma types and membership into each depression symptom trajectory group (n = 1,844). ^a^

|  | Crude | | | Adjusted | | |
| --- | --- | --- | --- | --- | --- | --- |
|  | OR | 95% CI | | OR | 95% CI | |
| Symptom-free group (reference) |  |  |  |  |  |  |
| Decreasing group, 1 trauma type | 2.13 | (1.50, 3.03) | | 2.09 | (1.47, 2.98) | |
| Decreasing group, 2 or more trauma types | 2.87 | (1.92, 4.31) | | 2.75 | (1.83, 4.13) | |
| Increasing group, 1 trauma type | 1.77 | (1.20, 2.61) | | 1.74 | (1.182, 2.57) | |
| Increasing group, 2 or more trauma types | 1.89 | (1.17, 3.04) | | 1.83 | (1.13, 2.95) | |
| Chronic group, 1 trauma type | 2.52 | (1.61, 3.94) | | 2.42 | (1.54, 3.8) | |
| Chronic group, 2 or more trauma types | 5.89 | (3.82, 9.07) | | 5.32 | (3.43, 8.27) | |

^a^ OR = odds ratio.

CI = confidence interval.

Controlling for biological sex, age group, and self-reported race and ethnicity. One participant was missing on number of types of childhood traumas and was excluded from this model. Exposure reference group = no trauma types.

Appendix Table 4. Fit statistics for latent class growth analysis of number of depression symptoms in the past 30 days, modeled using a zero-inflated Poisson distribution with different numbers of groups and function forms, among main cohort respondents with no history of depression at the start of follow-up (n = 880). ^a^

Appendix Table 4a.

|  | Functional form (0=intercept only, 1=linear, 2=quadratic, 3=cubic) | | | | |  |
| --- | --- | --- | --- | --- | --- | --- |
| Number of groups | Group 1 | Group 2 | Group 3 | Group 4 | Group 5 | BIC |
| 2 | 3 | 3 |  |  |  | -2795.19 |
| 2 | 3 | 2 |  |  |  | -2792.62 |
| 2 | 2 | 2 |  |  |  | -2790.37 |
| 2* | 1 | 2 |  |  |  | -2787.44 |
| 3 | 3 | 3 | 3 |  |  | -2671.55 |
| 3 | 2 | 3 | 3 |  |  | -2671.00 |
| 3 | 2 | 2 | 3 |  |  | -2665.74 |
| 3 | 1 | 2 | 3 |  |  | -2662.35 |
| 3 | 0 | 2 | 3 |  |  | -2660.21 |
| 3** | 0 | 2 | 2 |  |  | -2658.42 |
| 4 | 3 | 3 | 3 | 3 |  | -2621.72 |
| 4 | 2 | 3 | 3 | 3 |  | -2618.33 |
| 4 | 2 | 2 | 3 | 3 |  | -2615.08 |
| 4 | 1 | 2 | 3 | 3 |  | -2611.95 |
| 4 | 0 | 2 | 3 | 3 |  | -2608.73 |
| 4** | 0 | 2 | 3 | 2 |  | -2606.98 |
| 5 | 3 | 3 | 3 | 3 | 3 | -2591.07 |
| 5 | 3 | 3 | 3 | 2 | 3 | -2652.33 |
| 5 | 3 | 2 | 3 | 2 | 3 | -2609.43 |
| 5 | 2 | 2 | 3 | 2 | 3 | -2597.37 |
| 5 | 2 | 1 | 3 | 2 | 3 | -2591.68 |
| 5 | 2 | 1 | 2 | 2 | 3 | -2591.94 |
| 5 | 2 | 1 | 2 | 2 | 2 | -2586.9 |
| 5* | 2 | 0 | 2 | 2 | 2 | -2583.78 |

^a^ BIC = Bayesian Information Criterion. Smaller absolute values of BIC indicate better fit.

* = best model for the number of groups.

** = best overall model/chosen number of groups.

Appendix Table 4b.

| Number of groups | Average predicted probability of group 1 | Average predicted probability of group 2 | Average predicted probability of group 3 | Average predicted probability of group 4 | Average predicted probability of group 5 |
| --- | --- | --- | --- | --- | --- |
| 2* | 0.983 | 0.948 |  |  |  |
| 3** | 0.933 | 0.901 | 0.939 |  |  |
| 4** | 0.921 | 0.830 | 0.847 | 0.923 |  |
| 5* | 0.871 | 0.906 | 0.819 | 0.849 | 0.918 |

* = best model for the number of groups.

** = best overall model/chosen number of groups.

Appendix Figure 2. Latent trajectories for number of depression symptoms in the past 30 days across four follow-up years, modeling using a zero-inflated Poisson distribution, among main cohort respondents with no history of depression at the start of follow-up (n = 880), three-group model. ^a^


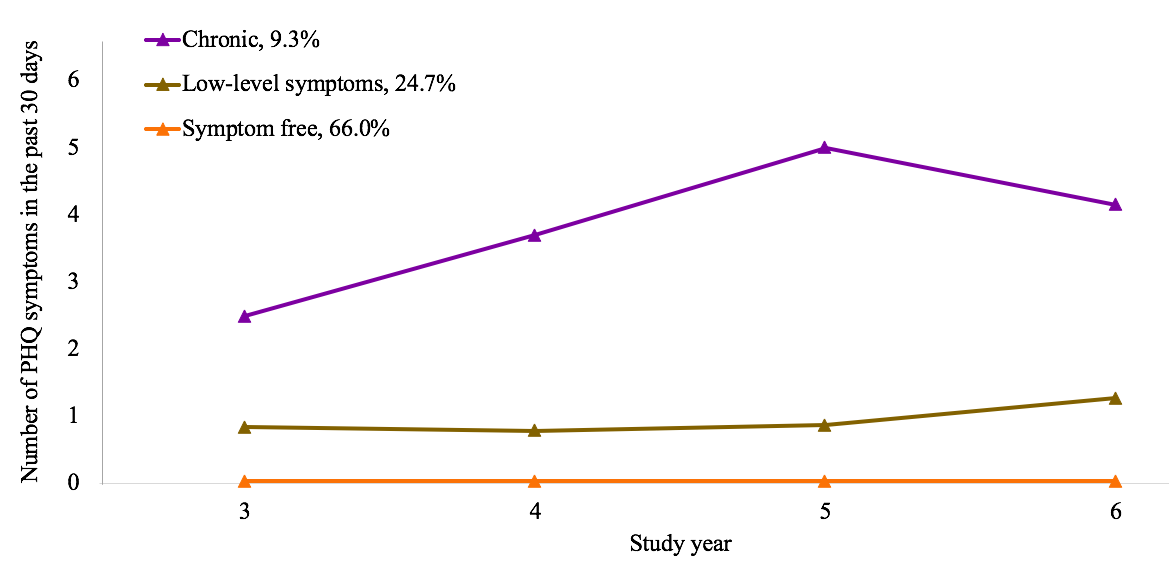


^a^ PHQ = Patient Health Questionnaire (nine total symptoms).

Appendix Figure 3. Latent trajectories for number of depression symptoms in the past 30 days across four follow-up years, modeling using a zero-inflated Poisson distribution, among main cohort respondents with no history of depression at the start of follow-up (n = 880), four-group model. ^a^


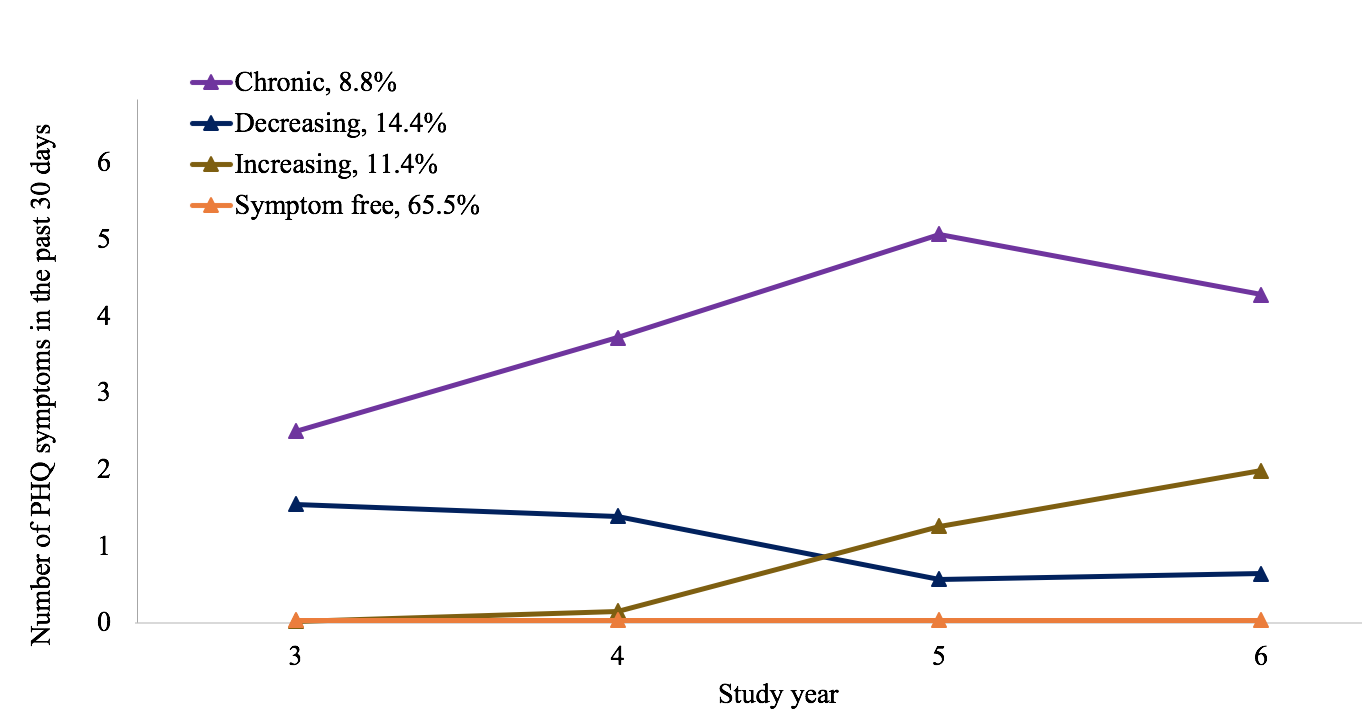


^a^ PHQ = Patient Health Questionnaire (nine total symptoms).

Appendix Table 5. Crude and fully adjusted multinomial models for the associations between reporting one or more traumatic childhood events and membership into each depression symptom trajectory group, among main cohort respondents with no history of depression at the start of follow-up (n = 880). ^a^

|  | Crude | | | Adjusted | | |
| --- | --- | --- | --- | --- | --- | --- |
|  | OR | 95% CI | | OR | 95% CI | |
| Symptom free group (reference) |  |  |  |  |  |  |
| Decreasing group | 1.45 | (0.89, | 2.34) | 1.49 | (0.91, | 2.44) |
| Increasing group | 1.87 | (1.13, | 3.11) | 1.89 | (1.14, | 3.16) |
| Chronic group | 2.25 | (1.30, | 3.88) | 2.15 | (1.24, | 3.73) |

^a^ OR = odds ratio.

CI = confidence interval.

Controlling for biological sex, age group, and self-reported race and ethnicity.

Appendix Figure 4. Latent trajectories for number of depression symptoms in the past 30 days across four follow-up years, modeled using a zero-inflated Poisson distribution, with and without past-year stressors at each follow-up year, among main cohort respondents with no history of depression at the start of follow-up (n = 880). ^a^


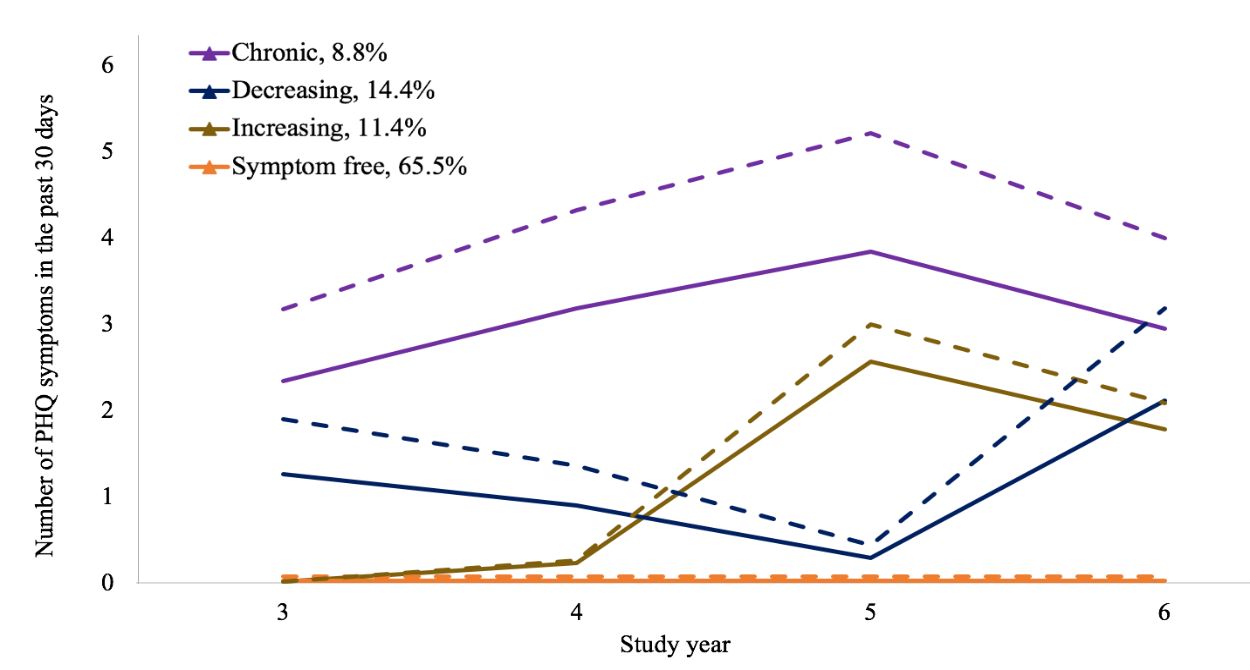


^a^ Dotted line = stressor at every time point, no PTSD at any time point.

Solid line = no stressors or PTSD at any time point.

PHQ = Patient Health Questionnaire (nine total symptoms).

Appendix Table 6. Crude odds ratios for the relationship between traumatic childhood events and membership into decreasing and chronic depression symptom trajectory groups compared to symptom free groups, after correcting for potential differential recall of traumatic childhood events by group membership. ^a^

|  | OR for decreasing group vs. symptom free group (n=1,431) | OR for chronic group vs. symptom free group (n=1,304) |
| --- | --- | --- |
| Uncorrected | 2.41 | 3.82 |
| Se, depression+: 0.90  Se, depression-: 0.80  Sp, depression+: 0.99  Sp, depression-: 0.99 | 2.21 | 3.67 |
| Se, depression+: 0.90  Se, depression-: 0.75  Sp, depression+: 0.99  Sp, depression-: 0.99 | 2.04 | 3.37 |
| Se, depression+: 0.90  Se, depression-: 0.80  Sp, depression+: 0.95  Sp, depression-: 0.99 | 1.94 | 3.33 |
| Se, depression+: 0.90  Se, depression-: 0.70  Sp, depression+: 0.99  Sp, depression-: 0.99 | 1.86 | 3.08 |
| Se, depression+: 0.90  Se, depression-: 0.70  Sp, depression+: 0.90  Sp, depression-: 0.95 | 1.78 | 3.24 |
| Se, depression+: 0.90  Se, depression-: 0.75  Sp, depression+: 0.95  Sp, depression-: 0.99 | 1.78 | 3.06 |
| Se, depression+: 0.90  Se, depression-: 0.70  Sp, depression+: 0.95  Sp, depression-: 0.99 | 1.63 | 2.80 |

^a^ OR = odds ratio.

Se = sensitivity.

Sp = specificity.

“+” = higher-symptom depression group; “-” = symptom free group.
